# Migration increases mitochondrial oxidative capacity without increasing reactive oxygen species emission in a songbird

**DOI:** 10.1242/jeb.246849

**Published:** 2024-05-10

**Authors:** Soren Z. Coulson, Christopher G. Guglielmo, James F. Staples

**Affiliations:** ^1^ Department of Biology, Western University, London, ON, Canada, N6A 5B7; ^2^ Centre for Animals on the Move, Western University, London, ON, Canada, N6A 3K7

**Keywords:** Flight muscle, Locomotory performance, OXPHOS, Endurance locomotion, Plasticity, ROS, Energetics

## Abstract

Birds remodel their flight muscle metabolism prior to migration to meet the physiological demands of migratory flight, including increases in both oxidative capacity and defence against reactive oxygen species. The degree of plasticity mediated by changes in these mitochondrial properties is poorly understood but may be explained by two non-mutually exclusive hypotheses: variation in mitochondrial quantity or in individual mitochondrial function. We tested these hypotheses using yellow-rumped warblers (*Setophaga coronata*), a Nearctic songbird which biannually migrates 2000–5000 km. We predicted higher flight muscle mitochondrial abundance and substrate oxidative capacity, and decreased reactive oxygen species emission in migratory warblers captured during autumn migration compared with a short-day photoperiod-induced non-migratory phenotype. We assessed mitochondrial abundance via citrate synthase activity and assessed isolated mitochondrial function using high-resolution fluororespirometry. We found 60% higher tissue citrate synthase activity in the migratory phenotype, indicating higher mitochondrial abundance. We also found 70% higher State 3 respiration (expressed per unit citrate synthase) in mitochondria from migratory warblers when oxidizing palmitoylcarnitine, but similar H_2_O_2_ emission rates between phenotypes. By contrast, non-phosphorylating respiration was higher and H_2_O_2_ emission rates were lower in the migratory phenotype. However, flux through electron transport system complexes I–IV, II–IV and IV was similar between phenotypes. In support of our hypotheses, these data suggest that flight muscle mitochondrial abundance and function are seasonally remodelled in migratory songbirds to increase tissue oxidative capacity without increasing reactive oxygen species formation.

## Teaser

**Summary:** Flight muscle mitochondria from migratory yellow-rumped warblers are more abundant, have higher phosphorylating respiration but similar reactive oxygen species emission compared with those of wintering warblers.

## INTRODUCTION

The mitochondrion is a dynamic organelle that is plastic in form and function in response to changes in cellular energy demands. This mode of plasticity allows animals to modulate cellular energy supply to maintain performance despite variation in energy demand. One activity of high energy demand is locomotion, and performance in this activity has a strong determining effect on predator evasion and foraging success. Chronic increases in locomotory activity elicit profound changes in mitochondrial physiology. For example, endurance exercise training increases muscle mitochondrial abundance in humans (Fernström et al., 2004; Meinild Lundby et al., 2018; Tonkonogi et al., 2000), rats (Davies et al., 1981; Galbès et al., 2008; Krieger et al., 1980; Zoll et al., 2003) (but see Farhat et al., 2015) and mice (Beyfuss et al., 2018; Caffin et al., 2013; Heo et al., 2021; Pastore and Hood, 2013; Southern et al., 2017). These increases in mitochondrial abundance typically result in greater oxidative phosphorylation (OXPHOS) capacity when measured in permeabilized muscle fibres of humans (Meinild Lundby et al., 2018; Pesta et al., 2011), rats (Zoll et al., 2003) and mice (Heo et al., 2021). These changes in whole-tissue metabolism are partly explained by changes in function of individual mitochondria. Specifically, endurance exercise training increases phosphorylating respiration in isolated muscle mitochondria in humans (Fernström et al., 2004; Tonkonogi et al., 2000) and mice (Southern et al., 2017), but not in rats (Davies et al., 1981; Galbès et al., 2008). As a result, training can increase whole-tissue oxidative capacity through increases in mitochondrial abundance and improved mitochondrial performance, depending on the species. Together, these changes proximately improve the capacity for ATP synthesis via oxidative phosphorylation and ultimately improve endurance exercise performance.

While mitochondrial changes in response to endurance exercise training are well defined, it remains poorly understood whether mitochondria respond similarly to increases in locomotion in animals that have been selected for endurance locomotion over many generations. Artificial selection for high activity induces elevated activity of mitochondrial enzymes in locomotor muscles in captive lab mice (Guderley et al., 2006; Houle-Leroy et al., 2003; Templeman et al., 2012), although most of these differences may be explained by variation in training volume (Houle-Leroy et al., 2000). It remains unclear how natural selection for high locomotory activity may affect mitochondrial physiology in wild animals, or in animals where locomotory activity changes seasonally.

Migratory birds provide an excellent model to study adaptations for endurance locomotion because of the use of long-distance movements between spatially disparate breeding and non-breeding grounds as part of their annual life-history cycle. These movements are performed via flight and allow birds to exploit spatiotemporal variation in resource availability. Aspects of migratory performance including timing and speed of migration are directly linked to reproductive performance via access to high-quality breeding territories, as shown in American redstarts (Smith and Moore, 2005). As a result, migratory performance, and its underlying physiological determinants, are under natural selection in songbirds. For all its potential benefits, migration is a significant challenge for birds because migratory flight is associated with the greatest movement requirements across the annual cycle, as shown in red-backed shrikes (Macías-Torres et al., 2022). Furthermore, migratory flight is a highly demanding mode of locomotion that requires metabolic rates approximately 12-fold higher than at rest and may be sustained continuously for several hours to days at a time (reviewed by Guglielmo, 2018). These high rates of oxidative metabolism are also associated with increased reactive oxygen species (ROS) formation during flight, which may oxidatively damage tissues and potentially compromise tissue function if ROS formation exceeds antioxidant capacity (Jenni-Eiermann et al., 2014). However, migratory birds have acquired a suite of physiological adaptations in the flight muscle to overcome these demands, including high capacities for fatty acid oxidation (reviewed by Guglielmo, 2018), made possible by elevated oxidative capacity (Lundgren, 1988) and a higher degree of coupling between mitochondrial respiration and oxidative phosphorylation (Toews et al., 2014) relative to non-migratory birds.

Daily energy expenditure during migration may be the highest across the annual cycle and 2-fold greater than in the wintering (i.e. non-breeding) season, as shown in lesser black-backed gulls (Brown et al., 2023). In response to this seasonal variation in energy demands, migratory birds have evolved an impressive degree of seasonal flexibility in flight muscle energy metabolism. Before migration, in the absence of training (Price et al., 2011), birds remodel the flight muscle to improve endurance flight performance via increased capacities for O_2_ and fuel delivery to mitochondria and consumption by mitochondria. These changes are achieved via reduced fibre diameter and increased capillary density (Ivy and Guglielmo, 2023), and increased expression of fatty acid binding protein and fatty acid translocase (Zajac et al., 2011; Zhang et al., 2015). At the mitochondrial level, flight muscle activity of β-hydroxyacyl-CoA dehydrogenase, citrate synthase and carnitine palmitoyl transferase can increase by 20–150%, 25–50% and 50–100%, respectively (Banerjee and Chaturvedi, 2016; Lundgren and Kiessling, 1986; Marsh, 1981; McFarlan et al., 2009; Sharma et al., 2021; Zajac et al., 2011) (but see DeMoranville et al., 2019; Price et al., 2010). However, it is not clear whether these changes simply reflect an increase in mitochondrial abundance or changes to mitochondrial function. Moreover, much less is known about seasonal flexibility in ROS management in the flight muscle of migratory songbirds, except to note that protein expression of Mn and Cu/Zn superoxide dismutases increases ∼1.2-fold prior to migration in red-headed buntings (Banerjee and Chaturvedi, 2016). Elevated capacities for ATP synthesis and ROS quenching may be beneficial for other phases of the annual life-history cycle beyond migration; however, birds transition out of the migratory phenotype following completion of migration. It is currently unclear why birds undergo this seasonal transition, but one potential explanation is the high physiological cost of maintaining the elevated total mitochondrial volume in flight muscle.

Migratory flight is fuelled largely by oxidation of fatty acids by flight muscle mitochondria (reviewed by Guglielmo, 2018), so we wanted to better understand how mitochondria respond seasonally in songbirds. In addition, the mitochondrial electron transport system (ETS) is a source of intracellular ROS production (reviewed by Hernansanz-Agustín and Enríquez, 2021). To our knowledge, no previous studies have examined seasonal variation in mitochondrial function in migratory birds. However, previous work has shown mixed evidence of seasonal mitochondrial plasticity in non-migratory songbirds that experience high wintering energy demands. Specifically, cold acclimation (which increases basal metabolic rate) has no effect on mitochondrial respiration in permeabilized pectoralis fibres from captive black-capped chickadees (Milbergue et al., 2022), while winter acclimatization increases leak respiration in red blood cell mitochondria in three species of Palearctic tits (Nord et al., 2021).

The objective of this study was to investigate how seasonal plasticity of flight muscle metabolism is mediated by changes in mitochondrial physiology in migratory songbirds. We hypothesized that mitochondria are seasonally plastic, and that their function would be altered in the migratory phenotype to support the physiological challenges of migratory flight. There are two non-mutually exclusive strategies to achieve this transition: (1) an increase in mitochondrial quantity and (2) an increase in mitochondrial quality. Under strategy 1, we predicted a higher pectoralis mitochondrial abundance in the migratory phenotype. Under strategy 2, we predicted that the migratory mitochondrial phenotype would include an elevated capacity for phosphorylating respiration and lower or similar rates of ROS formation, compared with the non-migratory phenotype. We tested these hypotheses by comparing the mitochondrial abundance and *ex vivo* function of flight muscle mitochondria from captive yellow-rumped warblers (*Setophaga coronata*) between migratory and non-migratory phenotypes. To our knowledge, this study is the first to characterize the seasonal transition between migratory and non-migratory phenotypes in songbird mitochondrial function.

## MATERIALS AND METHODS

### Experimental animals

All procedures followed guidelines set by the Canadian Council on Animal Care and were approved by the Western University Animal Care Sub-Committee (AUP 2018-092) and the Canadian Wildlife Service (SC-OR-2018-0256). We used a Nearctic migratory songbird, the myrtle subspecies of the yellow-rumped warbler, *Setophaga coronata coronata* (Linnaeus 1766). The myrtle warbler (hereafter ‘yellow-rumped warbler') is a 10–15 g wood warbler that breeds in the boreal forest throughout northern Canada and northeastern USA, and migrates to wintering grounds throughout southern USA to as far south as Panama (Hunt and Flaspohler, 1998). The high spatial variation in migration phenology is likely the result of facultative migration extension (Terrill and Ohmart, 1984) and generalist habitat requirements (Parnell, 1969).

Warblers were captured during the autumn migration season (2020) in mist nets at Long Point, ON, Canada (42.58°N, 80.40°W), a major stopover site for migrating songbirds. The age class and sex of each bird were determined from plumage and other characters, and wing chord was measured as a structural index of body size, as used previously in this and other songbird species (Kissner et al., 2003). We then transferred the birds into captivity at the Advanced Facility for Avian Research at Western University (London, ON, Canada) and housed them in aviaries (122×132×213 cm) at room temperature with *ad libitum* access to diet made in-house (Guglielmo et al., 2017) supplemented with mealworms (*Tenebrio molitor*) and water. We kept the birds on a long day photoperiod similar to natural conditions at Long Point (12.5 h:11.5 h light:dark) to preserve the migratory phenotype and randomly sampled half of the birds (*n*=8) within 2–4 weeks. We then manipulated the photoperiod to induce a non-migratory phenotype, as used previously in our lab for this species (Guglielmo et al., 2017) and other songbirds (Ivy and Guglielmo, 2023; Price et al., 2010, 2011). We progressively shortened the photoperiod for the remaining birds (*n*=8) by 1 h every 3 days until reaching a short-day photoperiod (9 h:15 h light:dark), then sampled the birds after 10–12 weeks. All birds were hatch-year, with similar sex ratios for each phenotype (migratory: 6 male, 2 unknown; non-migratory: 7 male, 1 unknown). These birds were also used for seasonal comparisons in pectoralis histology in a separate study (Ivy and Guglielmo, 2023). Our measurements in the non-migratory phenotype may be confounded by captivity-induced reduction in movement; however, the migratory phenotype in songbirds is largely determined by environmental factors (e.g. photoperiod), and the seasonal transition may be observed without accompanying changes in movement (Price et al., 2010, 2011; Zajac et al., 2011).

We humanely euthanized the birds using full anaesthesia under inhaled isofluorane followed by decapitation within 10 s. We weighed the bird, and then quickly removed and weighed the pectoralis major. A small piece (∼100 mg) from the middle of one side of the pectoralis was removed and snap-frozen in liquid nitrogen then stored at −80°C for enzyme analysis. We used the medial pectoralis to limit variation in enzyme activity across the pectoralis (Scott et al., 2009). We then lightly minced the remaining pectoralis in ice-cold biopsy preservation solution (Letellier et al., 1992; Veksler et al., 1987) (BIOPS; in mmol l^−1^: 2.77 CaK_2_EGTA, 7.23 K_2_EGTA, 5.77 Na_2_ATP, 6.56 MgCl_2_, 20 taurine, 15 Na_2_ phosphocreatine, 20 imidazole, 0.5 dithiothreitol, 50 MES, pH 7.1) and transferred the sample to the lab for mitochondrial isolation within 45 min.

### Mitochondrial isolation

We isolated pectoralis mitochondria using differential centrifugation methods described previously, with modifications (Gerson, 2012; Muleme et al., 2006). All procedures were performed at 4°C. We first washed the pectoralis twice with 10 ml of homogenization buffer (HB; in mmol l^−1^: 100 sucrose, 50 Tris, 10 EDTA, 100 KCl, pH 7.4) to remove BIOPS. We then finely minced and digested the pectoralis in 10 ml of HB with protease (Sigma P8038; 0.2 mg ml^−1^) for 3 min, stirring approximately once each minute. We decanted the HB and rinsed the pectoralis 3 times with 10 ml of HB. We homogenized the pectoralis using a loose-fitting Teflon pestle in a glass homogenizer at 100 rpm for 5–6 passes, or until no large pieces were visible, and filtered the homogenate through four layers of cheesecloth into a polycarbonate tube and centrifuged the filtrate at 1000 ***g*** for 10 min. We aspirated any lipids floating at the surface of the resulting supernatant and filtered the remaining supernatant through four layers of cheesecloth. We centrifuged this filtrate at 8700 ***g*** for 10 min and re-suspended the resulting pellet in HB. We repeated the 8700 ***g*** spin and re-suspended the resulting pellet in a small volume of HB (100–200 µl) as the crude mitochondrial extract. We determined mitochondrial protein concentration using a Bradford assay (Bradford, 1976) with bovine serum albumin (BSA) standards.

### Mitochondrial fluororespirometry

We measured mitochondrial respiration and ROS emission rates via high-resolution fluororespirometry using Clark-type polarographic oxygen electrodes and fluorometric sensors, respectively (Oxygraph-2k, Oroboros). Mitochondria were assayed at 39°C in 2 ml of respiratory buffer (Gnaiger et al., 2000) [in mmol l^−1^: 0.5 EGTA, 3 MgCl_2_, 20 taurine, 10 KH_2_PO_4_, 20 Hepes, 110 sucrose, 60 lactobionic acid; 0.1% (w/v) BSA, pH 7.1]. O_2_ electrodes were calibrated daily using respiratory buffer at air equilibration and in anoxia using a yeast suspension. To measure ROS emission rates, we used the fluorescence module (O2k-Fluo LED2) and tracked H_2_O_2_ emission via fluorescence linked to Amplex Ultra-Red (Makrecka-Kuka et al., 2015). Fluorometric sensors were calibrated for each sample after mitochondrial addition using two additions of freshly prepared 0.1 µmol l^−1^ H_2_O_2_ standards before and after each run. For all measurements, we waited for the O_2_ consumption rate to stabilize for at least 1 min.

We simultaneously measured respiration, ROS emission rates and O_2_ concentration during oxidation of pyruvate and palmitoylcarnitine in parallel. We first added 10 µmol l^−1^ Amplex Ultra-Red (Invitrogen), 1 U ml^−1^ horseradish peroxidase, 15 µmol l^−1^ DTPA and 5 U ml^−1^ superoxide dismutase to each chamber before addition of mitochondria (100 µg of mitochondrial protein). We added DTPA to reduce background fluorescence from contaminating iron, as reported previously (Komlódi et al., 2018). In one chamber, we stimulated oxidative phosphorylation with the addition of 1 mmol l^−1^ malate, 25 µmol l^−1^ palmitoylcarnitine and 200 µmol l^−1^ ADP to measure State 3 respiration. After, we added 25 nmol l^−1^ oligomycin to inhibit oxidative phosphorylation to measure State 4 respiration. We repeated these measurements in a separate chamber using 1 mmol l^−1^ pyruvate instead of palmitoylcarnitine. Respiration and ROS emission rates were corrected for background activity.

In a separate run, we measured mitochondrial respiration coupled to oxidative phosphorylation in response to stimulation by different electron sources. In duplicate, we added mitochondria and 0.5 mmol l^−1^ ADP. To assess the NADH (N) pathway (complex I–IV flux), we stimulated respiration by adding 1 mmol l^−1^ pyruvate and 1 mmol l^−1^ malate. Next, we inhibited complex I with 0.5 µmol l^−1^ rotenone and assessed the succinate (S) pathway (complex II–IV flux) by stimulating respiration with 5 mmol l^−1^ succinate. Finally, we measured complex IV-mediated respiration by inhibiting complex III with 25 nmol l^−1^ antimycin A and stimulating respiration with 0.5 mmol l^−1^ TMPD and 2 mmol l^−1^ ascorbate to limit TMPD auto-oxidation. To assess TMPD auto-oxidation, we re-oxygenated the chambers and measured respiration in the presence of 0.5 mmol l^−1^ KCN. We measured the difference in respiration between stimulation and inhibition of each complex as the complex-specific respiration. The remaining isolated mitochondrial preparations were frozen at −80°C for later analyses.

### Enzyme activity assays

We assessed the activity of four metabolic enzymes in the pectoralis to assess seasonal variation in fatty acid and glycolytic metabolism using methods described previously (Price et al., 2010). First, we finely minced the pectoralis (see above) with a razor blade on ice, then diluted the tissue 10-fold in homogenizing buffer (20 mmol l^−1^ Na_2_HPO_4_, 0.5 mmol l^−1^ EDTA, 0.2% BSA, 50% glycerol, 0.1% Triton X-100, 50 µg ml^−1^ aprotinin). We mechanically homogenized the pectoralis on low power for 3 bouts of 10 s, separated by 30 s rest on ice (Polytron PT 10-35, Kinematica, Bohemia, NY, USA). We then sonicated the homogenate on low power for 3 bouts of 10 s, separated by 30 s on ice (VirSonic 100, VirTis, Gardiner, NY, USA). Within an hour of homogenization, we measured the apparent maximal activity of carnitine palmitoyltransferase (CPT), citrate synthase (CS), lactate dehydrogenase (LDH) and β-hydroxyacyl-CoA dehydrogenase (HOAD) in pectoralis homogenate using a UV/Vis spectrophotometer at 39°C (Cary 100 Bio, Varian, Palo Alto, CA, USA). We assayed CPT and CS activity by measuring DTNB absorbance at 412 nm, and assayed HOAD and LDH activity by measuring NADH absorbance at 340 nm. We used the following assay conditions for each enzyme, with ‘±’ denoting the metabolite omitted for background activity measurement: (in mmol l^−1^) CPT: ±5 carnitine, 0.15 DTNB, 0.035 palmitoyl-CoA, 50 Tris (pH 8.0); CS: ±0.5 oxaloacetate, 0.15 DTNB, 0.15 acetyl-CoA, 50 Tris (pH 8.0); HOAD: ±0.1 acetoacetyl-CoA, 0.2 NADH, 1 EDTA, 50 imidazole (pH 7.4); LDH: ±4 pyruvate, 0.15 NADH, 5 dithiothreitol, 50 imidazole (pH 7.4). We assayed all enzymes in triplicate using 1.5 ml cuvettes at a final volume of 1 ml, corrected for background activity, and calculated enzyme activity using the extinction coefficients 1.36×10^4^ l mol^−1^ cm^−1^ (DTNB) and 6.22×10^3^ l mol^−1^ cm^−1^ (NADH). We measured pectoralis CS activity to infer variation in mitochondrial abundance. We also measured CS activity in the isolated mitochondrial preparations following one freeze–thaw cycle and sonication, using methods described above. In addition, we measured total antioxidant capacity in the isolated mitochondrial preparations using a commercially available kit that assesses enzymatic and non-enzymatic antioxidants via Cu^2+^ reduction (Abcam ab65329). Because of equipment failure on the day of measurement, we were only able to measure total antioxidant capacity in half (*n*=4) of the birds from each phenotype.

### Western blots

We used western blotting to assess variation in protein expression of ETS protein complexes. First, we isolated membrane proteins from the mitochondrial preparations used for respirometry. We pelleted the mitochondria at 15,000 ***g*** for 2 min at 4°C and incubated the re-suspended pellet in mitochondrial extraction buffer (in mmol l^−1^: 750 aminocaproic acid, 50 Bis-Tris, pH 7.0) with 3.5 g maltoside per gram protein on ice for 15 min. We then centrifuged these samples at 13,000 ***g*** for 30 min at 4°C and discarded the pellet. We quantified protein concentration in these extracts using a commercially available bicinchoninic acid assay (Thermo Scientific 23225). We diluted the samples in Laemmli buffer (10% glycerol, 40 mmol l^−1^ Tris, 2% SDS, 0.005% Bromophenol Blue, 1% β-mercaptoethanol) and boiled them for 5 min using a hot water bath.

We then performed SDS-PAGE using pre-cast 10% polyacrylamide gels in the Bio-Rad mini-PROTEAN system (Bio-Rad, Hercules, CA, USA). Samples and protein ladder (Bio-Rad 161-0373) were loaded onto the gel with running buffer (192 mmol l^−1^ glycine, 25 mmol l^−1^ Tris, 0.1% SDS; pH 8.3) and run at 50 V for 10 min, followed by 120 V for 55 min. We wet-transferred the samples onto polyvinylidene difluoride membrane in transfer buffer (20% methanol, 192 mmol l^−1^ glycine, 25 mmol l^−1^ Tris) at 4°C by running at 100 V for 2 h, then washed the membrane 3 times for 5 min each with TBS-T (0.05% TWEEN-20, 20 mmol l^−1^ Tris, 144 mmol l^−1^ NaCl) and blocked the membrane overnight in TBS-T with 5% BSA at 4°C. We then immersed the membrane in 5% BSA with a cocktail of mouse primary antibodies for each of the ETS protein complexes (Abcam ab110413) diluted 1:1000, followed by incubation for 2 h at room temperature. We washed the membrane in TBS-T for 10 min repeated 3 times, immersed the membrane in donkey anti-mouse secondary antibody (Abcam ab6820) diluted 1:2000 in TBS-T and incubated it at room temperature for 1 h. We repeated the membrane washes in TBS-T and imaged the blots using the Bio-Rad Clarity Western ECL substrate system in a Bio-Rad Chemi-Doc Touch imaging system.

### Statistical analysis

All analyses were performed on R (version 4.3.1; http://www.R-project.org/) using the *car* package (https://cran.r-project.org/web/packages/car/index.html) for general linear modelling (Fox and Weisberg, 2019). We conducted general linear modelling to assess differences between phenotypes while controlling for covariation with body mass, except for pectoralis mass, which was compared after controlling for covariation with wing chord as a structural size measure. We took a similar approach to assess mitochondrial ROS emission, but included O_2_ concentration as an additional covariate (Li Puma et al., 2020). All covariates with *P*<0.1 were included in the final model. When no covariates were included, we used two sample, unpaired *t*-tests. We calculated free radical leak [FRL; 100×(ROS emission/(2×respiration)] to assess electron leak from the ETS as previously described (Brown et al., 2009). We also assessed the coupling of mitochondrial respiration to oxidative phosphorylation by calculating OXPHOS control efficiency (1−State 4 respiration/State 3 respiration) and respiratory control ratio (RCR; State 3/State 4 respiration) (Divakaruni and Jastroch, 2022). To assess respiratory capacity independent of H^+^ leak, we calculated net OXPHOS capacity (State 3 respiration−State 4 respiration) (Gnaiger, 2012). As both FRL and OXPHOS control efficiency are proportions, we arcsine square root-transformed them prior to statistical comparisons to ensure normality in our dataset. When linear modelling detected significant or nearly significant covariation, we calculated estimated marginal means using the *emmeans* package (https://CRAN.R-project.org/package=emmeans) and plotted these values to better show between-phenotype variation.

All data presented in the main text are available in Table S1; all data presented in the supplementary information are available in Table S2.

## RESULTS

### Body and tissue mass

**Fig. 1.**
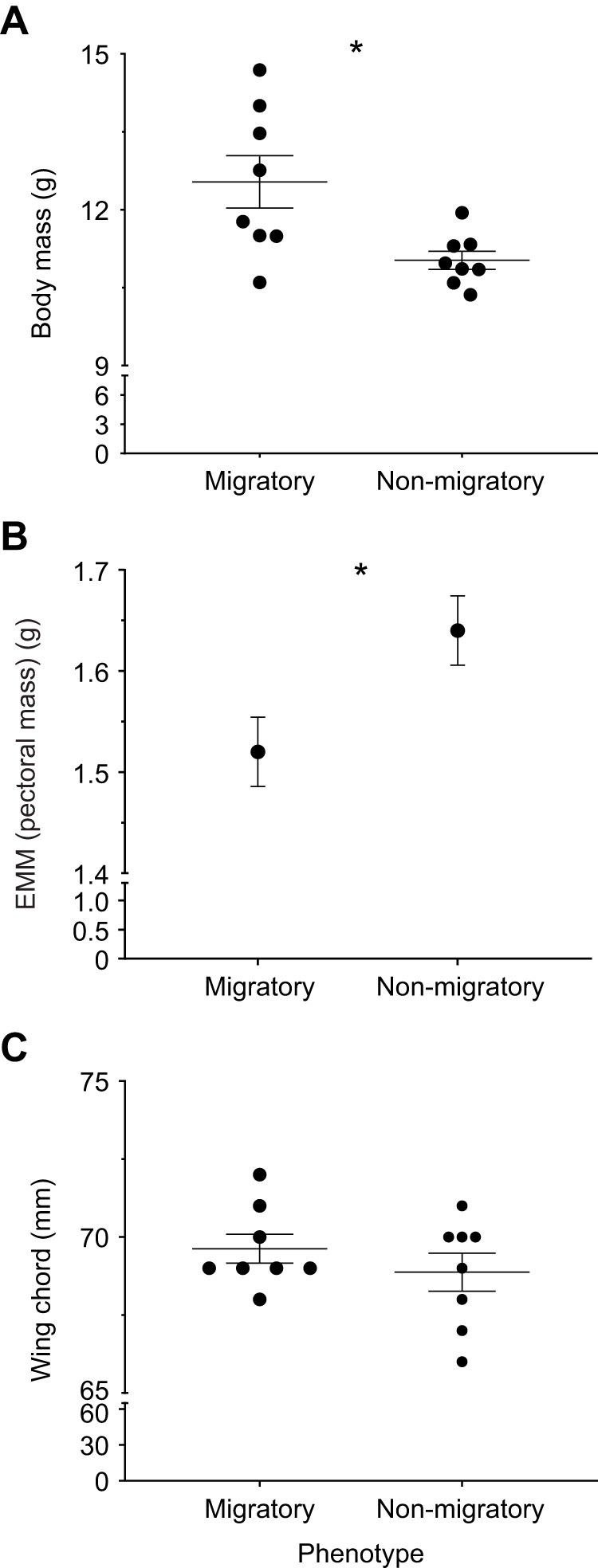
**Seasonal variation in body composition in yellow-rumped warblers.** Total body mass (A), estimated marginal means (EMM) of wet pectoralis mass, taking covariation from wing chord into effect (B) and wing chord (C) of migratory warblers sampled during autumn migration and non-migratory warblers sampled following short-day photoperiod acclimation. Data are means±s.e.m., *N*=8. Asterisks indicate a significant difference between phenotypes (*P<0.05).

The body mass of migratory warblers was ∼14% higher than that of non-migratory warblers (*t*_8.65_=2.83, *P*=0.02; Fig. 1A). The estimated marginal mean (EMM) for pectoralis mass was ∼8% higher in the non-migratory warblers (Fig. 1B; *F*_1,13_=6.1, *P*=0.03) with a nearly significant effect of wing chord (*F*_1,13_=3.9, *P*=0.07). Wing chord was similar between phenotypes (*t*_14_=0.98, *P*=0.34; Fig. 1C). In contrast, heart mass and liver mass did not correlate with wing chord; both heart mass (*t*_13.8_=0.0, *P*=1.00) and liver mass (*t*_8.0_=−0.5, *P*=0.62) were similar between phenotypes (Fig. S1).

### Enzyme activity assays

After accounting for variation in body mass (*F*_1,13_=3.8, *P*=0.07), we found 60% higher pectoralis CS activity in the migratory phenotype (Table 1; *F*_1,13_=13.1, *P*<0.01). Similarly, we found 46% and 71% higher activity in the migratory phenotype for CPT (Table 1; *t*_14.0_=2.18, *P*<0.05) and HOAD (Table 1; *t*_9.2_=3.46, *P*<0.01), respectively. In contrast, we found 39% lower LDH activity in the migratory phenotype (Table 1; *t*_14.0_=2.18, *P*<0.05). The above enzyme activity measurements are expressed per unit mass of pectoralis, but because of seasonal variation in pectoralis mass, we also calculated whole-organ activity. We found ∼30%, ∼50% and ∼60% higher whole-pectoralis activity for CS (*t*_14_=2.25, *P*=0.04), CPT (*t*_14_=1.89, *P*=0.08) and HOAD (*t*_9.0_=2.85, *P*=0.02), respectively, while whole-pectoralis LDH activity was ∼30% lower in the migratory phenotype (*t*_14_=2.33, *P*=0.03). CS activity of the isolated mitochondrial preparations (migratory: 1.48±0.66 µmol min^−1^ mg^−1^ protein, non-migratory: 1.56±1.07 µmol min^−1^ mg^−1^ protein) was similar between phenotypes (*t*_8.6_=1.24, *P*=0.25).

**Table 1.**
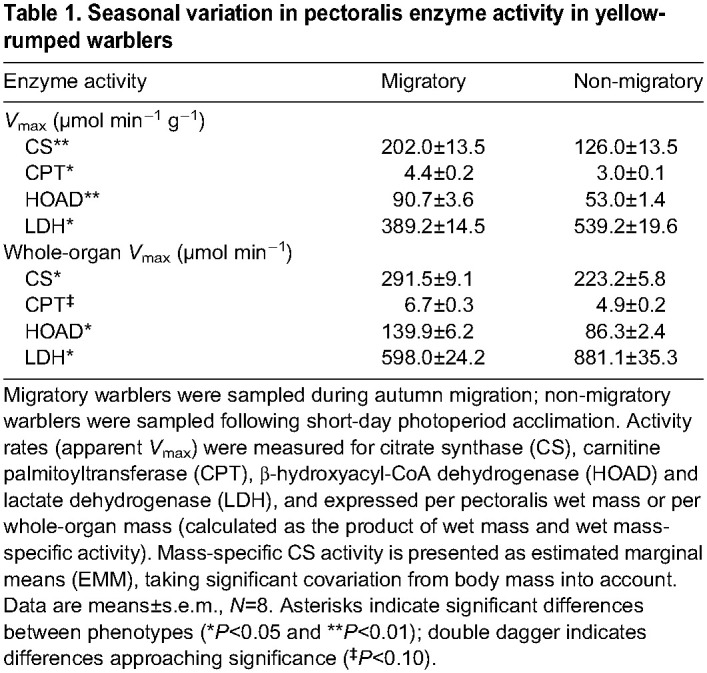
Seasonal variation in pectoralis enzyme activity in yellow-rumped warblers

### Mitochondrial substrate oxidation

**Fig. 2.**
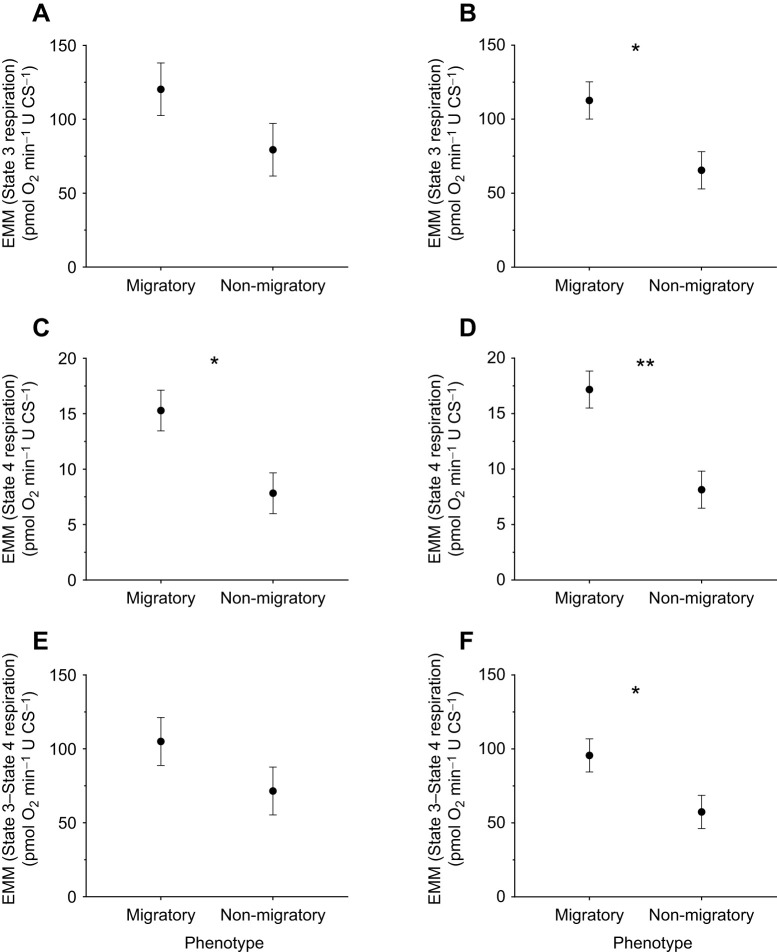
**Seasonal variation in substrate oxidation in yellow-rumped warbler pectoralis mitochondria.** O_2_ consumption rates (EMM after accounting for covariation from body mass) of isolated pectoralis mitochondria from migratory and non-migratory warblers, measured during oxidation of pyruvate (left) or palmitoylcarnitine (right) substrate in phosphorylating (State 3; A,B) or non-phosphorylating conditions (State 4; C,D), and net oxidative phosphorylation (OXPHOS) capacity presented as the difference between State 3 and State 4 respiration (E,F). O_2_ consumption rates are expressed relative to citrate synthase (CS) activity (µmol min^−1^; U) of individual mitochondrial preparations. Data are means±s.e.m., *N*=8. Asterisks indicate significant differences between phenotypes (*P<0.05 and **P<0.01).

We measured phosphorylating (State 3) and non-phosphorylating (State 4) respiration for isolated pectoralis mitochondria during oxidation of two different substrates (Fig. 2). Because mitochondrial CS activity did not differ between phenotypes, we express respiration relative to CS activity, but respiration rates relative to mitochondrial protein are shown in Fig. S2. We found similar State 3 pyruvate respiration between phenotypes (Fig. 2A; *F*_1,13_=2.2, *P*=0.16) after accounting for the effects of body mass (*F*_1,13_=3.8, *P*=0.07). In contrast, State 3 palmitoylcarnitine respiration was ∼70% higher in the migratory phenotype (Fig. 2B; *F*_1,13_=5.7, *P*=0.03) after correcting for body mass (*F*_1,13_=7.0, *P*=0.02). We also found ∼95% higher State 4 pyruvate respiration in the migratory phenotype (Fig. 2C; *F*_1,13_=6.7, *P*=0.02) after accounting for body mass (*F*_1,13_=3.3, *P*=0.09). Similarly, we found that State 4 palmitoylcarnitine respiration was 110% higher in the migratory phenotype (Fig. 2D; *F*_1,13_=12.0, *P*<0.01) after accounting for body mass (*F*_1,13_=7.7, *P*=0.02). Despite this increase in State 4 respiration, net OXPHOS capacity was ∼65% higher in the migratory phenotype (Fig. 2F; *F*_1,13_=4.8, *P*<0.05) when oxidizing palmitoylcarnitine, and after accounting for body mass (*F*_1,13_=6.7, *P*=0.02). However, after accounting for body mass (*F*_1,13_=3.7, *P*=0.08), we found similar net OXPHOS capacity between phenotypes for pyruvate oxidation (Fig. 2E; *F*_1,13_=1.7, *P*=0.21). In contrast, when we expressed respiration relative to mitochondrial protein, we found little variation between phenotypes in State 3 respiration (*t*_10.9_=−1.4, *P*=0.20), State 4 respiration (*t*_12.5_<0.1, *P*=1.0) and net OXPHOS capacity (*t*_10.8_=−1.5, *P*=0.16) when oxidizing pyruvate (Fig. S2). Similarly, after expressing respiration relative to mitochondrial protein, we found little phenotypic variation in State 3 respiration (*t*_14_=−0.6, *P*=0.56), State 4 respiration (*t*_14.0_=0.5, *P*=0.63) and net OXPHOS capacity (*t*_14.0_=−0.76, *P*=0.46) when oxidizing palmitoylcarnitine.

**Fig. 3.**
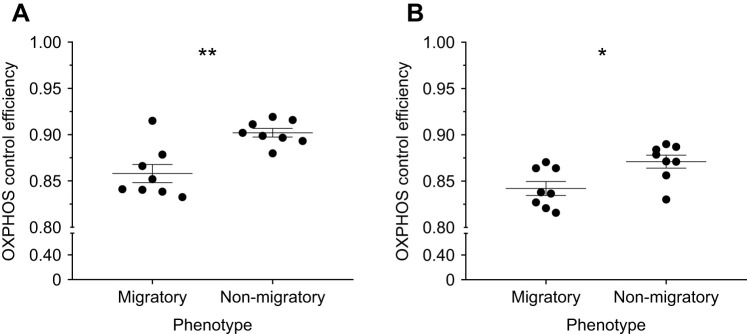
**Seasonal variation in coupling of mitochondrial respiration to oxidative phosphorylation in yellow-rumped warblers.** O_2_ consumption rates of isolated pectoralis mitochondria from migratory and non-migratory warblers, measured during oxidation of pyruvate (A) or palmitoylcarnitine (B) substrate in phosphorylating and non-phosphorylating conditions. OXPHOS control efficiency was calculated as the proportion of State 3 O_2_ consumption linked to oxidative phosphorylation. Data are means±s.e.m., *N*=8. Asterisks indicate significant differences between phenotypes (*P<0.05 and **P<0.01).

To better assess coupling between respiration and oxidative phosphorylation, we calculated OXPHOS control efficiency (Fig. 3) and RCR (Fig. S3). We found 5% and 4% lower OXPHOS control efficiency in the migratory phenotype for pyruvate (Fig. 3A; *t*_10.5_=4.0, *P*<0.01) and palmitoylcarnitine (Fig. 3B; *t*_14.0_=2.9, *P*=0.01), respectively. Similarly, we found 27.5% and 18.5% lower RCR in the migratory phenotype for pyruvate (*t*_12.4_=3.3, *P*<0.01) and palmitoylcarnitine (*t*_13.6_=3.0, *P*=0.01), respectively.

### NADH and succinate pathway flux

**Fig. 4.**
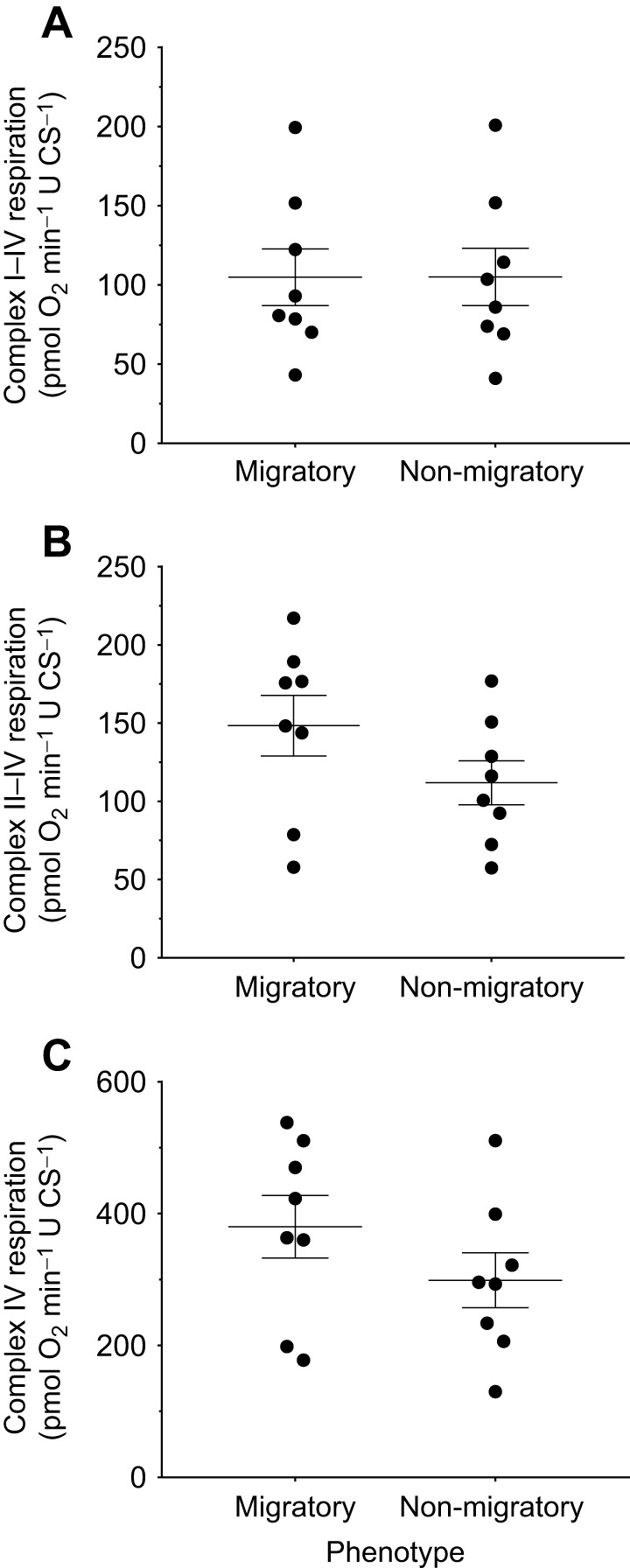
**Seasonal variation in electron transport system complex-specific flux in isolated pectoralis mitochondria from yellow-rumped warblers.** Respiration rates specific to (A) complex I–IV, (B) complex II–IV and (C) complex IV, expressed relative to CS activity (µmol min^−1^; U) of individual mitochondrial preparations from migratory and non-migratory warblers. Data are means±s.e.m., *N*=8.

To better understand variation in State 3 respiration, we measured phosphorylating respiration via the NADH and succinate pathways, mediated by complexes I–IV and II–IV, respectively, and complex IV capacity (Fig. 4). We found similar respiration rates between phenotypes for the NADH pathway (Fig. 4A; *t*_14_<0.1, *P*=0.99), the succinate pathway (Fig. 4B; *t*_14_=1.5, *P*=0.15) and complex IV capacity (Fig. 4C; *t*_14_=1.29, *P*=0.22). Similarly, when expressed per mitochondrial protein (Fig. S4), respiration rates were similar between phenotypes for the NADH pathway (*t*_13.5_=1.4, *P*=0.18), succinate pathway (*t*_12.8_=0.23, *P*=0.82) and complex IV capacity (*t*_12.0_=0.38, *P*=0.71).

### Mitochondrial ROS emission

**Fig. 5.**
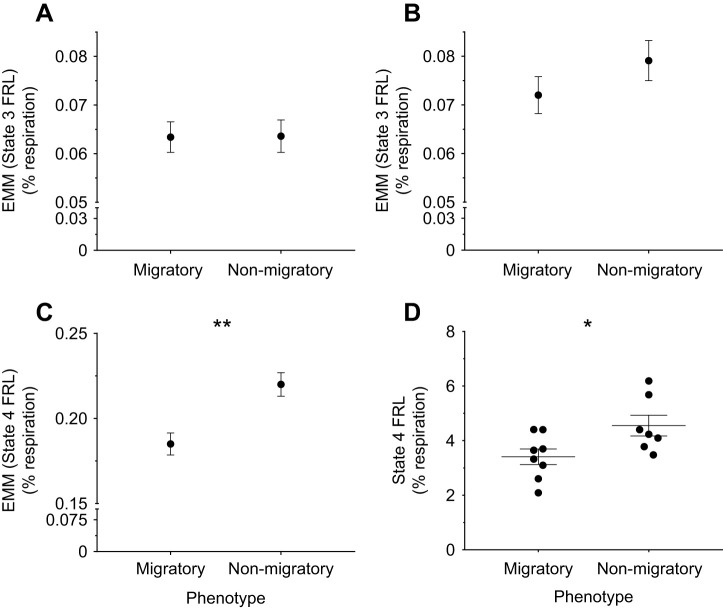
**Seasonal variation in mitochondrial free radical leak in yellow-rumped warbler pectoralis mitochondria.** Free radical leak (FRL) of isolated pectoralis mitochondria from migratory and non-migratory warblers, measured during oxidation of pyruvate (left) or palmitoylcarnitine (right) substrate in phosphorylating (State 3; A,B) or non-phosphorylating (State 4; C,D) conditions. FRL was calculated as H_2_O_2_ emission rate standardized to atoms of oxygen consumed and expressed as a percentage of oxygen consumed. FRL is presented as EMM taking covariation from assay O_2_ concentration (A,C) or body mass (B) into effect. Data are means±s.e.m., *N*=8 migratory, *N*=7 non-migratory. Asterisks indicate significant differences between phenotypes (*P<0.05 and **P<0.01).

We removed data from one non-migratory bird from our dataset as an outlier for each ROS measurement. We found similar State 3 FRL between phenotypes for pyruvate (Fig. 5A; *F*_1,12_<0.01, *P*=0.96), which positively correlated with assay O_2_ concentration (*F*_1,12_=5.14, *P*=0.04). State 3 FRL was also similar between phenotypes for palmitoylcarnitine (Fig. 5B; *F*_1,12_=1.29, *P*=0.28), with nearly significant covariation with body mass (*F*_1,12_=3.59, *P*=0.08). In contrast, we found that State 4 FRL was ∼16% lower in the migratory phenotype for pyruvate (Fig. 5C; *F*_1,12_=13.35, *P*<0.01) after accounting for assay O_2_ concentration (*F*_1,12_=7.29, *P*=0.02). Similarly, State 4 FRL was ∼20% lower for palmitoylcarnitine in the migratory phenotype (Fig. 5D; *t*_14_=2.96, *P*=0.01). We also found similar mitochondrial total antioxidant capacities (migratory: 0.62±0.15 pmol Trolox U CS^−1^, non-migratory: 0.50±0.15 pmol Trolox U CS^−1^) between phenotypes (*t*_6_=0.61, *P*=0.57). We also present absolute ROS emission rates standardized to CS activity and mitochondrial protein in Table 2. When standardized to CS activity, absolute ROS emission rates were similar between phenotypes for palmitoylcarnitine oxidation in State 3 (*t*_13_=0.19, *P*=0.85) and State 4 respiration (*t*_13_=0.19, *P*=0.85). ROS emission rates per CS activity were also similar between phenotypes for pyruvate in State 3 respiration (*F*_1,11_=0.87, *P*=0.37), after accounting for near-significant covariation with body mass (*F*_1,11_=4.2, *P*=0.07) and significant covariation with O_2_ concentration (*F*_1,11_=5.3, *P*=0.04). Similarly, State 4 ROS emission for pyruvate did not vary between phenotypes (*F*_1,11_=1.85, *P*=0.20, but covariation was nearly significant with body mass (*F*_1,11_=4.6, *P*= 0.06) and significant with O_2_ concentration (*F*_1,11_=5.0, *P*<0.05). In contrast, ROS emission rates standardized to mitochondrial protein were ∼35% and ∼30% lower in the migratory phenotype for State 3 (*t*_13_=3.14, *P*<0.01) and State 4 respiration (*t*_13_=3.38, *P*<0.01) for pyruvate, respectively. ROS emission rates per mitochondrial protein for palmitoylcarnitine were similar between phenotypes in State 3 respiration (*t*_9.0_=0.96, *P*=0.36) but were ∼25% lower in the migratory phenotype in State 4 respiration, a difference that approached significance (*t*_13_=1.88, *P*=0.08).

**Table 2.**
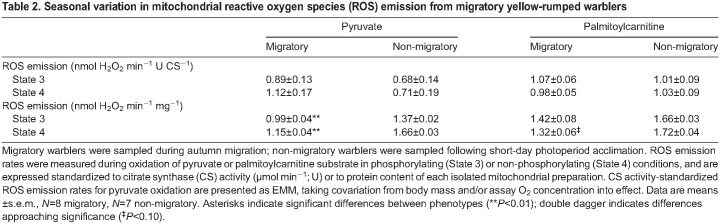
Seasonal variation in mitochondrial reactive oxygen species (ROS) emission from migratory yellow-rumped warblers

### Western blots

To assess seasonal potential variation in ETS protein complex abundance as a mechanistic explanation for variation in State 3 respiration, we conducted western blotting using a cocktail containing one primary antibody that cross-reacts with one peptide from each of the ETS complexes in mice and other rodents, as previously used in our lab (Mathers and Staples, 2019). Preliminary experiments revealed that this antibody cocktail produced five bands in proteins extracted from rat gastrocnemius mitochondria, but only two bands in flight muscle mitochondria isolated from blackpoll warblers (*Setophaga striata*), ruffs (*Calidris pugnax*) and yellow-rumped warblers (Fig. S5). These bands were approximately 54 and 34 kDa, which we interpret to correspond to peptides from complexes V and II, respectively, based on similarity in mass with bands from rat mitochondria. Bands corresponding to complex I, III and IV peptides were not visible in any gels from the three bird species, suggesting that avian epitopes do not cross-react with these mammalian-derived antibodies. Nonetheless, we found greater density of bands consistent with complexes V (migratory: 4.1 a.u. µg^−1^ protein; non-migratory: 2.31 a.u. µg^−1^ protein) and II (migratory: 2.96 a.u. µg^−1^ protein; non-migratory: 1.89 a.u. µg^−1^ protein, where a.u. is arbitrary units) in the migratory phenotype.

## DISCUSSION

We tested the hypotheses that flight muscle mitochondria are seasonally plastic, and that mitochondrial function is altered in the migratory phenotype to better meet the challenges of migratory flight. In support of both hypotheses, in the migratory phenotype we found higher pectoralis CS activity indicating a greater mitochondrial abundance. In isolated mitochondria, we also found higher State 3 respiration fuelled by palmitoylcarnitine and lower State 4 FRL. Taken together, these results indicate that yellow-rumped warblers seasonally modulate mitochondrial quantity and mitochondrial function to increase the capacity for energy provisioning while reducing oxidative costs of ROS during migration.

The two groups of birds used in our study were held in captivity for different durations, and it is possible that this contributed to the differences we found, perhaps through captivity stress. However, we believe this is unlikely because all birds in this study were healthy and exhibited normal behaviour, and previous work in this species has shown limited effects of time in captivity on body composition (Dick and Guglielmo, 2019). Furthermore, seasonal flight muscle plasticity follows similar patterns in birds sampled in the wild in different seasons (McFarlan et al., 2009) and in those sampled in captivity under different photoperiods (Zajac et al., 2011), as shown in white-throated sparrows. We believe, therefore, that our results represent a real effect of the migratory phenotype.

Our data indicate a high degree of seasonal plasticity in flight muscle metabolism in migratory birds. We found higher mass-specific activity of mitochondrial enzymes (CS, HOAD, CPT) and lower activity of the glycolytic enzyme LDH in the migratory phenotype, indicating that the migratory pectoralis phenotype has an elevated capacity for aerobic metabolism, in accord with previous seasonal comparisons in this species (Dick, 2017) and other songbirds (Banerjee and Chaturvedi, 2016; Lundgren and Kiessling, 1986; Marsh, 1981; McFarlan et al., 2009; Sharma et al., 2021; Zajac et al., 2011). Surprisingly, we found that the pectoralis was smaller in the migratory phenotype, in contrast to previous findings of pectoralis hypertrophy during migration in songbirds (DeMoranville et al., 2019; Price et al., 2011). The lower pectoralis mass in the migratory phenotype may be partly explained by a lower muscle fibre diameter in these birds (Ivy and Guglielmo, 2023) and by a lower proportion of fast-glycolytic muscle fibres (Chang et al., 2024). These data may reflect a lower capacity for mechanical power output in the migratory phenotype for yellow-rumped warblers. Nonetheless, when we expand the mass-specific enzyme activity to whole-organ activity, we still find higher activity of CS, HOAD and CPT in the migratory phenotype, reflecting an overall greater oxidative capacity, which would help power endurance flight. Furthermore, maintaining a high cellular oxidative capacity may allow yellow-rumped warblers to minimize pectoralis size and reduce flight costs via reduced body mass.

In mammals, a muscle phenotype similar to that of migratory yellow-rumped warblers has been identified in ‘mini mice’ that have evolved smaller locomotory muscles with higher mass-specific mitochondrial enzyme activity in response to artificial selection for high running activity (Guderley et al., 2006; Houle-Leroy et al., 2003; Templeman et al., 2012). While these mice required ∼11–12 weeks of wheel access to attain this phenotype, yellow-rumped warblers attain similar results following cessation of breeding, potentially without increasing locomotory activity. However, total muscle activity of mitochondrial enzymes is similar between mini-mice and control mice lines (Houle-Leroy et al., 2003; Templeman et al., 2012), while total flight muscle activity is greater in migratory than in non-migratory warblers. As a result, yellow-rumped warblers may be able to simultaneously modulate muscle size and oxidative capacity in response to increased locomotory demands, while lab mice may not share this plasticity.

We infer higher mitochondrial abundance in the migratory phenotype from higher CS activity, but we recognize that CS activity has not been validated as a proxy for mitochondrial volume by microscopy in birds, as has been done with endurance exercise training in humans (Meinild Lundby et al., 2018). However, transmission electron microscopy has revealed increases in flight muscle mitochondrial volume during migration in shorebirds (Evans et al., 1992). A seasonal increase in pectoralis mitochondrial volume could result from upregulation in signalling from perixosome proliferator-activated receptor γ co-activator-1α (PGC-1α) and downstream increased expression of nuclear receptor factors 1 and 2 (reviewed by Jornayvaz and Shulman, 2010). Seasonal variation in PGC-1α signalling is poorly understood in migratory songbirds, but mRNA expression of the functionally similar PGC-1β increases 2.2-fold during migration in yellow-rumped warblers (Dick, 2017). In contrast, PGC-1α mRNA expression is ∼70% higher during autumn migration than during the breeding season, with few changes in PGC-1β in gray catbirds, despite the absence of seasonal changes in mitochondrial abundance (DeMoranville et al., 2019). Future investigations should explore the physiological processes underlying the seasonal changes in pectoralis mitochondrial abundance.

These whole-tissue changes are supported by seasonal remodelling of individual mitochondria, as State 3 respiration and net OXPHOS capacity for palmitoylcarnitine oxidation were higher during migration. This greater capacity for fatty acid oxidation may be required to offset the potential costs of greater uncoupling of substrate oxidation from ADP phosphorylation during migration (Fig. 3; Fig. S3), which may reduce the efficiency of ATP synthesis. In addition, an increase in total mitochondrial abundance may further offset greater uncoupling by increasing tissue oxidative capacity. Future studies in seasonal variation in mitochondrial function of migratory birds should directly assess mitochondrial ATP synthesis relative to O_2_ consumption and ROS emission to provide a more holistic view of mitochondrial performance.

We found lower State 4 FRL in mitochondria from the migratory phenotype, suggesting that these mitochondria emit fewer ROS at submaximal workloads. These conditions correspond to high membrane potential, where ROS formation is likely near maximal. Nonetheless, absolute ROS emission rates (per mg protein) were lower in the migratory phenotype for both State 3 and State 4 respiration. We also found similar total mitochondrial antioxidant capacity between phenotypes, suggesting that the lower ROS emission in migration is not due to higher rates of ROS quenching, and instead may be the result of lower ETS electron leak (Murphy, 2009). However, to better understand *in vivo* mitochondrial ROS dynamics, future studies should address seasonal variation in the kinetics of mitochondrial ROS emission and quenching, as has been performed in rat skeletal muscle (Treberg et al., 2019). A lower ETS electron leak can be explained by a relatively more oxidized ETS, particularly at complexes I or III, where electrons can ‘leak’ from the ETS onto O_2_ to form O_2_·^−^ (Hernansanz-Agustín and Enríquez, 2021). Net decreases in ETS reduction state may be caused by increases in ‘spare capacity’ of the ETS via higher activity/abundance of ETS complexes, or by uncoupling ETS flux from OXPHOS (Brown et al., 2009; Lane, 2005). Both mechanisms divert electrons away from the principal sources of electron leak (Murphy, 2009). It remains unclear whether migratory birds increase ETS ‘spare capacity’ during migration, but our findings of higher State 4 respiration and lower OXPHOS coupling efficiency during migration both indicate an increase in uncoupling of respiration from ATP synthesis. ETS electron leak may also be reduced by the increased formation of ETS supercomplexes, which may decrease reduction state by increasing electron flux among ETS complexes (Maranzana et al., 2013). While such supercomplexes increase in formation with exercise training in humans (Greggio et al., 2017), it is presently unclear whether supercomplex abundance is seasonally plastic in migratory birds. A lower ETS electron leak is likely beneficial for migrating birds by reducing the potential for oxidative damage to intracellular proteins, membranes and free fatty acids.

Our observations of higher State 4 respiration rates in the migratory phenotype suggest higher H^+^ permeability across the inner mitochondrial membrane; however, this should be interpreted cautiously, as our study did not control for membrane potential, which partly determines H^+^ conductance (Divakaruni and Brand, 2011). A higher H^+^ permeability may be explained by increased inner mitochondrial membrane phospholipid fatty acyl polyunsaturation (Brand et al., 2003) and/or by the increased abundance of anion carrier proteins, such as adenine nucleotide translocase and uncoupling proteins (reviewed by Divakaruni and Brand, 2011). It is presently unclear whether mitochondrial membrane composition is seasonally altered in migratory birds, but polyunsaturated phospholipid composition of whole flight muscle does change seasonally in white-throated sparrows (Klaiman et al., 2009), though not in white-crowned sparrows (Price et al., 2010). Flight muscle phospholipid fatty acyl polyunsaturation is sensitive to dietary changes in songbirds, with turnover of most fatty acids taking ∼2–3 weeks (Carter et al., 2019; Dick and Guglielmo, 2019; Price and Guglielmo, 2009). Our migratory birds were sampled within 2–4 weeks of capture, potentially before complete membrane turnover would occur. However, this confounding effect is likely negligible because the majority of H^+^ conductance across the inner mitochondrial membrane can be explained by expression of adenine nucleotide translocase (Brand et al., 2005). Seasonal expression patterns of adenine nucleotide translocase and avian uncoupling protein in migratory songbirds are presently unknown and are thus exciting avenues for future research.

Despite apparent advantages of the migratory phenotype (greater fatty acid oxidation, reduced ROS emission), this phenotype is not expressed year-round in songbirds. Our findings of an increased State 4 respiration in tandem with higher flight muscle mitochondrial abundance suggest an elevated energetic burden of maintaining the migratory phenotype. Leak respiration of liver and skeletal muscle mitochondria is estimated to account for ∼15–20% of whole-animal standard metabolic rate in mammals (Rolfe and Brand, 1996; Rolfe et al., 1999) but, unfortunately, comparable data for birds do not exist, to our knowledge. Moreover, data on seasonal flexibility in basal metabolic rate in migratory birds are limited and contradictory (McKechnie, 2008). However, the lower pectoralis mass in the migratory phenotype in our study may compensate for a higher per-unit mass metabolic cost of maintaining this tissue. Nonetheless, our observations of increased State 4 respiration in the migratory phenotype agree with other observations. For example, whole-animal maximal metabolic rate induced by cold in black-capped chickadees (Milbergue et al., 2022) and exercise in brown trout (Salin et al., 2016), correlate positively with mitochondrial State 4 respiration, although mechanistic explanations underlying this relationship are unclear. As a result, elevated State 4 respiration may accompany the elevated whole-animal maximal metabolic rate previously observed during migration in songbirds (DeMoranville et al., 2019; Swanson, 1995; Swanson and Dean, 1999).

Together, these data suggest that flight muscle mitochondria are seasonally remodelled to increase cellular oxidative capacity while reducing the potential for oxidative stress. Both seasonal changes are likely to benefit migrating songbirds by proximately increasing ATP supply while reducing oxidative damage to the flight muscle, which may ultimately translate to improved migration speed by avoiding compromised flight performance and prolonged recovery times at stopover (McWilliams et al., 2021).

## Supplementary Material

10.1242/jexbio.246849_sup1 Supplementary information

Table S1. Data presented in main text

Table S2. Data presented in supplementary information
