# Supplementary material for: Migration increases mitochondrial oxidative capacity without increasing reactive oxygen species emission in a songbird
Source: J Exp Biol. 2024 May 10;227(9):jeb246849. doi: 10.1242/jeb.246849 (PMC11128287; doi:10.1242/jeb.246849)
Supplement: Supplementary information [file jexbio-227-246849-s1.pdf]

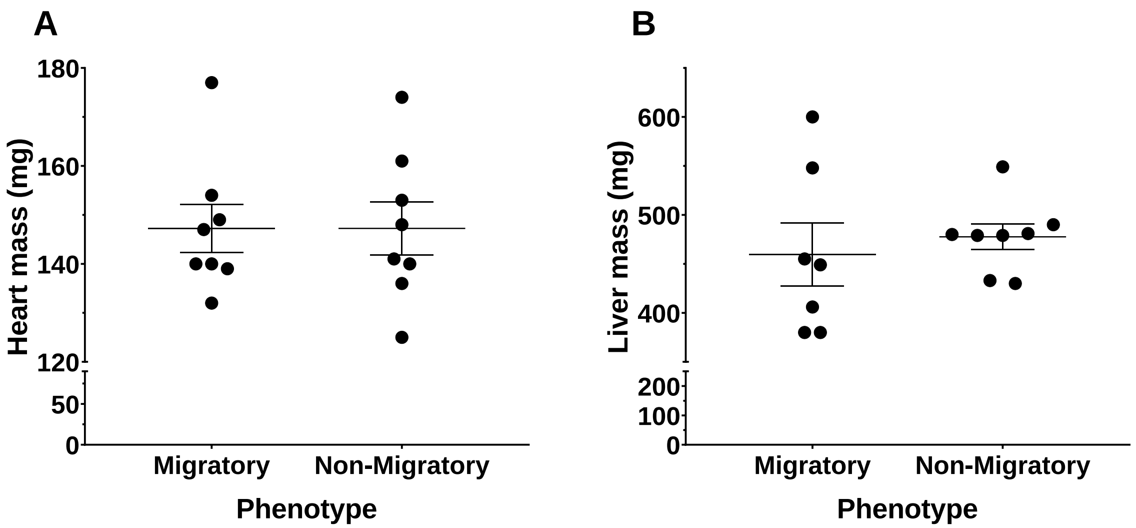

Fig. S1. Seasonal variation in organ mass in yellow-rumped warblers. Wet masses of heart (A) and liver (B). Migratory warblers sampled during autumn migration, non-migratory warblers sampled following short-day photoperiod acclimation. Data presented as mean ± s.e.m. N = 7-8.

Journal of Experimental Biology • Supplementary information

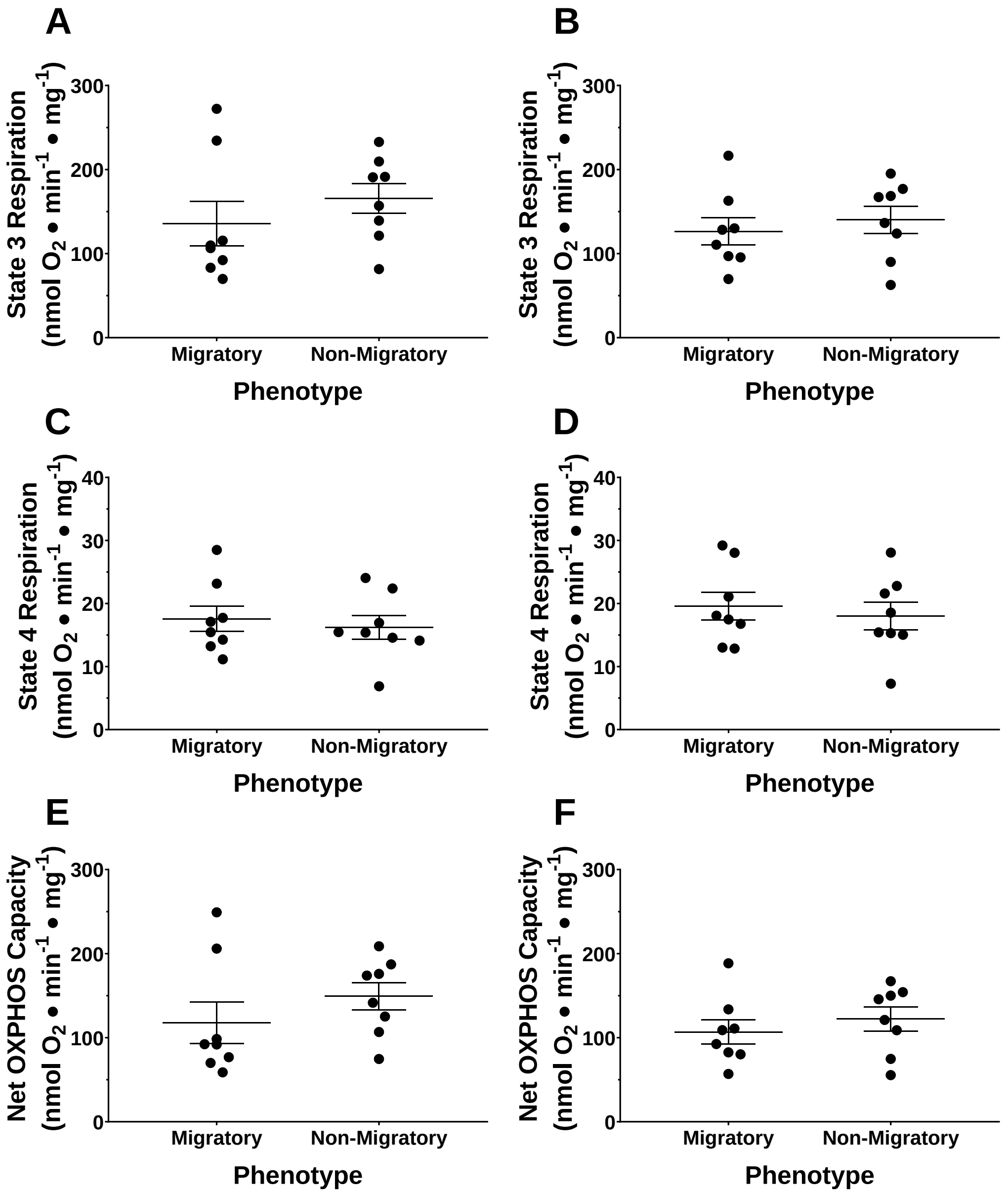

Fig. S2. Seasonal variation in substrate oxidation in yellow-rumped warbler pectoralis mitochondria. Respiration rates of isolated pectoralis mitochondria were measured during oxidation of pyruvate (A, C, E) or palmitoyl-carnitine (B, D, F) substrate in phosphorylating (State 3; A, B) or non-phosphorylating conditions (State 4; C, D). Net OXPHOS capacity calculated as difference between state 3 and state 4 respiration rates. Respiration rates are expressed relative to protein content of individual mitochondrial preparations. Migratory warblers sampled during autumn migration, non-migratory warblers sampled following short-day photoperiod acclimation. Data presented as mean ± SE. N = 8.

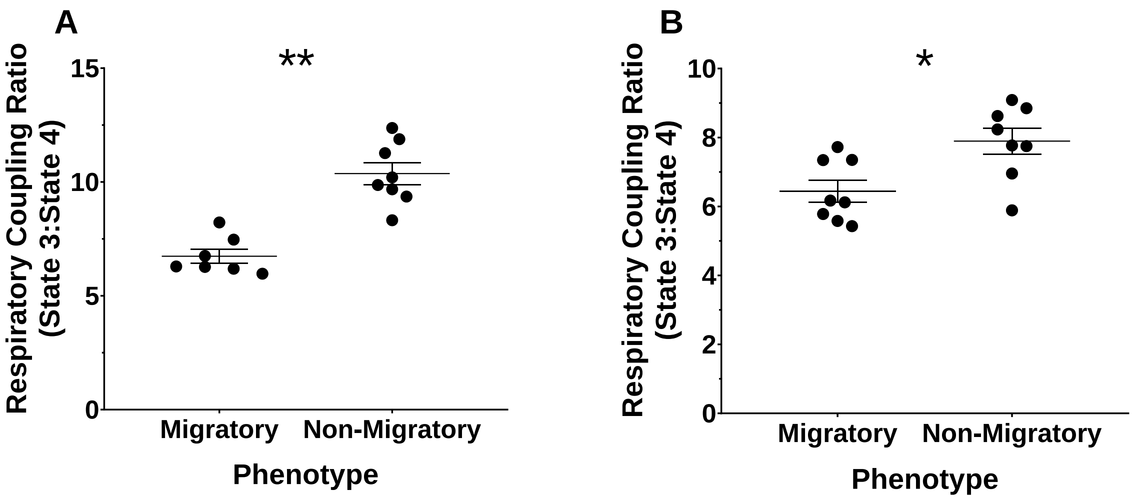

Fig. S3. Seasonal variation in coupling of mitochondrial respiration to oxidative phosphorylation in yellow-rumped warblers. Respiration rates of isolated pectoralis mitochondria were measured during oxidation of pyruvate (A) or palmitoyl-carnitine (B) substrate in phosphorylating (state 3) and non-phosphorylating (state 4) conditions. Respiratory control ratio was calculated as the ratio of state 3 respiration to state 4 respiration. Migratory warblers sampled during autumn migration, non-migratory warblers sampled following short-day photoperiod acclimation. Data presented as mean ± s.e.m. N = 7 (migratory), 8 (non-migratory). Significant differences between phenotypes indicated by * ($p < 0.05$) and ** ($p < 0.01$).

Journal of Experimental Biology • Supplementary information

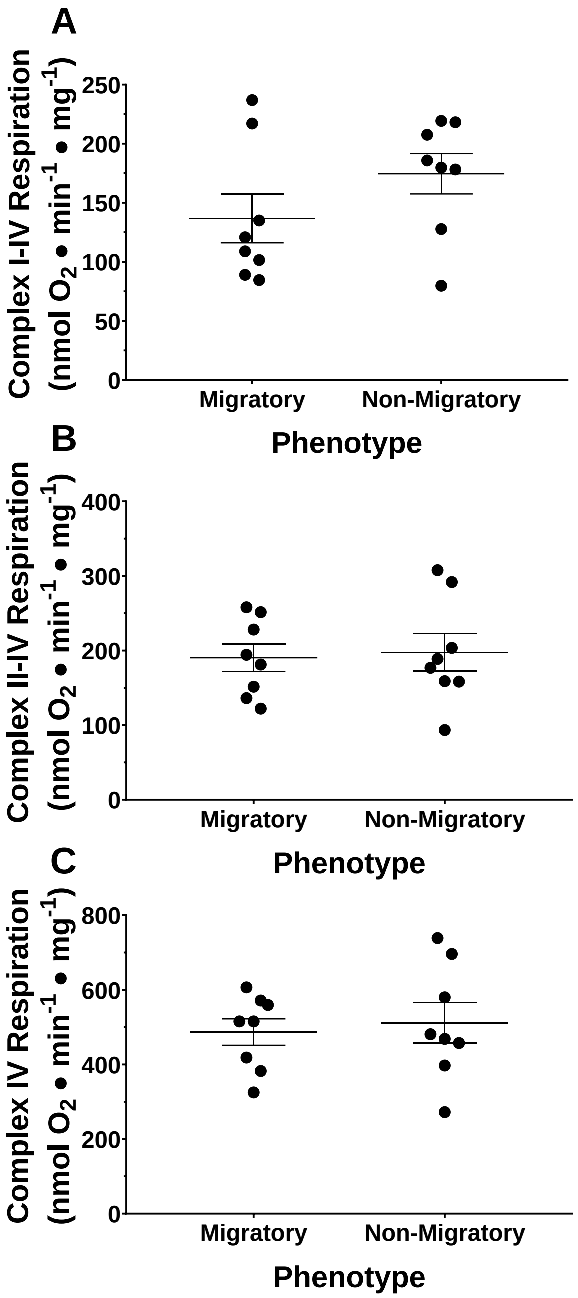

Fig. S4. Seasonal variation in electron transport system complex-specific flux in isolated pectoralis mitochondria from yellow-rumped warblers. Respiration rates specific to A) complex I-IV, B) complex II-IV and C) complex IV. Respiration rates expressed relative to protein content of individual mitochondrial preparations. Migratory warblers sampled during autumn migration, non-migratory warblers sampled following short-day photoperiod acclimation. Data presented as mean ± SE. N = 8.

Journal of Experimental Biology • Supplementary information

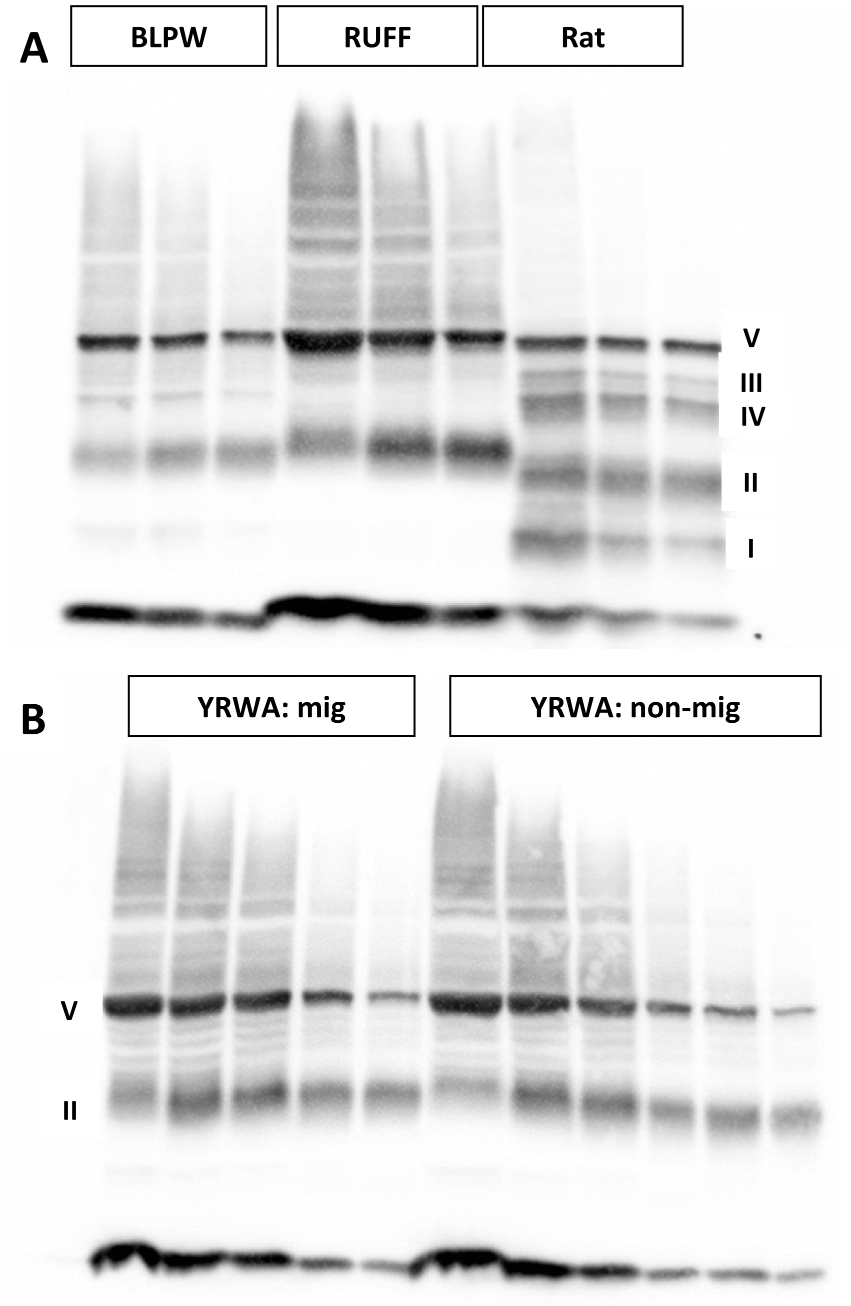

Fig. S5. Western blotting of mitochondrial electron transport system complexes. Blots for three concentrations from two-fold serial dilutions of blackpoll warblers (BLPW), ruffs (RUFF) and rat muscle mitochondria (A) and for 5-6 concentrations of yellow-rumped warbler (YRWA) flight muscle mitochondria from migratory and non-migratory phenotypes (B). Bands are labelled by corresponding putative electron transport system complex.

Journal of Experimental Biology • Supplementary information

Table S1. Data presented in main text

Available for download at
https://journals.biologists.com/jeb/article-lookup/doi/10.1242/jeb.246849#supplementary-data

Table S2. Data presented in supplementary information

Available for download at
https://journals.biologists.com/jeb/article-lookup/doi/10.1242/jeb.246849#supplementary-data
